# Efficacy and Safety of Endoscopic Ultrasound-Guided Drainage in Liver Abscess Management: A Systematic Review and Meta-Analysis

**DOI:** 10.1055/a-2919-1534

**Published:** 2026-07-29

**Authors:** Spyridon Peppas, Farhan Kawsar, Jerapas Thongpiya, Tara Keihanian, Jordan Sparkman, Salmaan Jawaid, Wasif Abidi, Kalpesh Patel, Mohamed O. Othman, Fares Ayoub

**Affiliations:** 1 Section of Gastroenterology, Hepatology & Nutrition 3989 Baylor College of MedicineHoustonTexasUnited States; 2 Internal Medicine 3989 Baylor College of MedicineHoustonTexasUnited States

**Keywords:** endoscopy upper GI tract, malignant strictures, endoscopic resection (ESD, EMRc, …), pancreatobiliary (ERCP/PTCD)

## Abstract

**Purpose** Percutaneous drainage is the standard nonsurgical treatment for liver abscess. Recent retrospective studies suggest that endoscopic ultrasound (EUS)-guided drainage may be an effective and safe alternative. We conducted a systematic review and meta-analysis to evaluate the efficacy and safety of EUS-guided drainage as a stand-alone approach and in comparison, with percutaneous drainage.

**Methods** PubMed, EMBASE, and Cochrane databases were searched from inception through November 2025. The primary outcome was clinical success; secondary outcomes included technical success, total adverse events, and abscess relapse. Sensitivity analyses using a treatment-arm continuity correction were performed to address zero-event cells.

**Results** Eleven studies were included (EUS, *n* = 186; percutaneous drainage, *n* = 325). In single-arm studies, pooled technical and clinical success rates with EUS were both 1.00 (95% CI, 0.95–1.00 and 0.99–1.00, respectively). Pooled total adverse event and relapse rates were 0.02 (95% CI, 0.00–0.12) and 0.00 (95% CI, 0.00–0.01), respectively. Comparative studies showed no significant differences between EUS and percutaneous drainage in technical success (RR 1.00, 95% CI, 0.83–1.19) or clinical success (RR 1.02, 95% CI, 0.86–1.22). EUS was associated with a significantly lower risk of total adverse events (RR 0.34, 95% CI, 0.15–0.76), while relapse rates were similar (RR 0.83, 95% CI, 0.30–2.33). Sensitivity analyses yielded consistent findings across all outcomes.

**Conclusion** EUS-guided drainage is an effective and safe treatment for liver abscesses primarily involving the left hepatic and caudate lobes. Although EUS was associated with a lower risk for adverse events, larger comparative studies are needed to define its role in right lobe abscesses.

## 1 Introduction

Hepatic abscesses are focal, well-circumscribed collections of purulent material resulting from mostly bacterial, but occasionally parasitic and fungal infections. 1 Although relatively uncommon in high-resourced settings, they still remain a clinically significant entity given the substantial morbidity and mortality associated with delays in diagnosis and source control. 2 The most common mechanism by which hepatic abscesses occur is via an underlying biliary tract infection (i.e., ascending cholangitis) followed by local extension and portal seeding. 3 Although the majority of the patients with liver abscess are treated conservatively, source control with drainage is critically important in management of hepatic abscesses >5 cm in size, multiloculated fluid collections, impending rupture, and no response to conservative management. 4 Traditionally, percutaneous catheter drainage (PCD) has served as the first-line interventional approach for image-guided drainage due to its minimal invasiveness and high rates of technical success. 5

However, PCD complications can include local pain and discomfort, peri-catheter leakage, bleeding, accidental dislodgement, and infections. 6 EUS-guided liver abscess drainage (EUS-LAD) offers an attractive alternative to percutaneous option for these lesions by providing a shorter access route through the gastric or duodenal walls. EUS-guided drainage may reduce the need for long transhepatic catheterization, avoid risks of pleural or pulmonary transgression, and eliminate complications related to retention of an external catheter. 7 EUS-LAD typically utilizes lumen-apposing metallic stents (LAMS), fully vs. partially covered self-expandable metal stents (FCSEMS vs PCSEMS) and double pigtail plastic stents to achieve adequate transmural drainage. 8 Additionally, in order to prevent the risk of stent migration, novel PCSEMS with an anti-migratory system have been developed and used in clinical practice. 9 10 In the existing literature, most studies have evaluated the drainage of abscesses in the left hepatic or caudate lobe with the EUS approach, although emerging evidence suggests that with recent advances, drainage of right hepatic lobe fluid collections can be technically feasible. 8

Although adoption remains slow in Western centers, EUS-LAD has demonstrated favorable short- and long-term outcomes. The aim of this meta-analysis is to provide a high-level, comprehensive overview of the current literature evaluating the safety and efficacy of EUS-guided liver abscess drainage, both as a standalone approach and in comparison with percutaneous needle aspiration or catheter drainage.

## 2 Methods

This review followed the Preferred Reporting Items for Systematic Reviews and Meta-Analyses (PRISMA) guidelines. 11 The study protocol was prospectively registered in PROSPERO (CRD420251266507).

### 2.1 Search Strategy

We conducted a comprehensive search of observational and randomized controlled studies evaluating the efficacy and safety of EUS-guided drainage of liver abscess in PubMed, Embase, and Cochrane databases from inception to November 30, 2025. The search strategy combined the terms “endoscopic ultrasound”, “EUS”, “EUS-guided drainage”, “EUS-guided”, “EUS guided”, “liver abscess”, “hepatic abscess”, “liver infected collection”, and “hepatic infected collection”. Editorials, notes, commentaries, correspondences, reviews, and nonhuman studies were excluded. Additionally, we manually screened the reference lists of relevant systematic reviews if available. No language restrictions were applied.

### 2.2 Study Selection and Outcomes

We included observational studies and randomized controlled trials, including conference abstracts, that evaluated the efficacy and safety of EUS-guided drainage of liver abscesses as a single-arm strategy, but also in comparison with percutaneous drainage. Thus, we considered eligible single-arm studies assessing EUS-guided drainage as a stand-alone approach and studies comparing EUS with percutaneous drainage if available. Given the emerging role of EUS-guided drainage in the management of liver abscesses, small case series enrolling at least three patients were considered eligible. Studies involving pediatric populations or those that did not report outcomes of interest were excluded. Two reviewers (SP, FK) independently screened titles and abstracts, and potentially eligible studies were subsequently reviewed in full text. Discrepancies were resolved through discussion and, when necessary, by consultation with an additional team member (JT).

The primary outcome studied in our meta-analysis was clinical success. Technical success was defined as the successful deployment of stent, access, and drainage of the liver abscess. Clinical success was described as complete resolution of clinical symptoms (e.g., fever, abdominal pain) with at least 50% reduction of the size of the abscess on follow-up cross-sectional imaging. However, given the novelty of the procedure and the expectation that available evidence would be limited to small case series, we included studies with alternative definition of clinical success to increase the number of eligible studies.

Secondary outcomes included the technical success, total adverse event rate and abscess relapse rate associated with the EUS-guided approach, as well as comparisons of technical and clinical success between EUS-guided and percutaneous drainage. Technical success was defined as the successful deployment of stent, access, and drainage of the liver abscess. Abscess relapse was defined as the recurrence of symptoms (e.g., abdominal pain, fever) combined with observation of liver abscess at the same site of prior lesion on cross-sectional imaging. Similar to the primary outcome of clinical success, we decided to include studies with alternative definitions to maximize the number of studies for the final analysis.

### 2.3 Data Extraction

Two reviewers (SP, FK) independently extracted data using a standardized electronic form. Extracted variables included study name, first author, year of publication, participant characteristics, sample size, age, sex, etiology and size of liver abscess, technical characteristics of the procedure (stent type and size), follow-up period, and number of events for each outcome of interest. These data were retrieved from the articles’ texts and supplementary materials.

### 2.4 Quality Assessment

The risk of bias was assessed by two independent investigators (SP, FK) by using the Risk of Bias in Non-randomized Studies – of Interventions, (ROBINS-I) tool for comparative studies, and the NIH Quality Assessment Tool for Before-After (Pre-Post) Studies with No Control Group for single-arm studies. 12 13 Each study was assessed for bias in the following domains: (a) confounding, (b) selection of participants, (c) classification of interventions, (d) departures from intended interventions, (e) missing data, (f) measurement of outcomes, and (g) selection of reported results. Included studied were categorized as “low”, “moderate”, “high”, or “critical” risk of bias. Any discrepancies in the quality assessment were resolved via consensus by a third author (JT).

### 2.5 Data Synthesis and Analysis

We calculated the weighted pool rates of technical and clinical success, and total adverse and relapse rates with their corresponding 95 % confidence intervals (CI) and *p* values. A *p* -value < 0.05 was considered statistically significant. For studies comparing the EUS-guided and percutaneous approach, risk ratios (RRs) with corresponding 95% confidence intervals were calculated. For studies with zero events in one or both arms, a continuity correction of 0.5 was applied. Sensitivity analyses using a treatment-arm continuity correction and risk difference as an alternative effect measure were performed to assess the robustness of ols. No adjustment for multiple comparisons was performed. Therefore, the reported confidence intervals and p-values should be interpreted without control of the overall Type I error rate across outcomes. The corresponding forest plots were generated for the graphical presentation of clinical outcomes. Heterogeneity between trials was quantified by measuring inconsistency using the Higgins *I* ^2^ index. 14 Studies were classified “low” ( *I* ^2^ < 25%), “moderate” ( *I* ^2^ 25–75%), or “substantial” ( *I* ^2^ > 75%) heterogeneity. Publication bias assessment would be derived only for outcomes involving ≥10 studies. 15 All analyses were conducted using the random effects model. STATA 19.0 was used as the statistical software for the analyses and generation of forest plots.

## 3 Results

### 3.1 Study Characteristics

A total of 511 unique records were identified, and 21 full-text articles were assessed for eligibility. Finally, 11 observational studies (7 single-arm and 4 comparative studies) fulfilled the eligibility criteria and were included in the meta-analysis **.** 7 16 17 18 19 20 21 22 23 24 25 **Fig. S1** summarizes the evidence search and selection (PRISMA flowchart). 26 Overall, the studies included 511 patients with 186 of them undergoing EUS-guided drainage of liver abscess and 325 patients undergoing a percutaneous approach. Four studies directly compared the two approaches in technical and clinical success, as well as their total adverse event rate. Most of the patients were males (EUS group, *n* = 132 patients, 73.7% vs. percutaneous group, *n* = 196 patients, 65.8%). Six studies indicated that the most common causes for undergoing an EUS approach compared to the percutaneous method were patient’s preference for an internal drainage, risk for self-tube removal due to underlying dementia, presence of ascites, and radiologically inaccessible site of the liver abscess. The etiology was reported in 9 studies, with the most common etiology being a pyogenic abscess in 229 patients (49.0%) followed by an amebic abscess in 57 patients (12.2%).

Considering the technical aspects of EUS, the most common approach included the stomach ( *n* = 94, 53.1%), followed by the duodenum ( *n* = 71, 40.1%), the esophagus ( *n* = 11, 6.2%), and the jejunum ( *n* = 1, 0.6%). Primary drainage of liver abscess was performed using a metal stent in 88 patients (36 with LAMS and 52 with FCSEMS or PCSEMS), a double pigtail plastic stent in 42 patients, a naso-cystic drainage tube in 39 patients, and single aspiration in 16 patients. Two studies reported that the placement of additional plastic stents for stabilization of metal stents and prevention of stent migration. 7 17

The baseline characteristics with the primary and secondary outcomes of included studies are summarized in Tables 1 and 2 . Additionally, definitions of the outcomes assessed from each study are reported in **Supplementary Table 1** .

**Table 1:** Baseline demographic and procedural data of included studies in the final analysis.

Study	Design	Country	No. of patients	Age (yrs)	Male sex, *N* (%)	Etiology of liver abscess, *N* (%)	Location of liver abscess, *N* (%)	Size of liver abscess (cm)	EUS approach, *N* (%)	Drainage method used on EUS group, *N* (%)	Procedure time
Shah 2023	Retrospective	India	EUS: 8	37.33 ± 12.8	5 (62.5)	Amebic: 5 (62.5) Infected pseudocyst: 2 (25.0) Pyogenic: 1 (12.5)	Caudate lobe: 8 (100.0)	5.76 ± 2.07	Transgastric 8 (100.0)	DP plastic: 6 (75.0) Aspiration: 1 (12.5) LAMS: 1 (12.5)	NR
Ogura 2016	Retrospective comparative	Japan	EUS: 8 PCD: 19	EUS: 66.5 (31–84) PCD: 66 (46–85)	EUS: 4 (50.0) PCD: 16 (84.2)	Pyogenic: 26 (96.3) Amebic: 1 (3.7)	EUS: Left 6 (75.0), Right 2 (25.0) PCD: Left 12 (63.2), Right 7 (36.8)	EUS: 7.46 (6.19–9.93) PCD: 7.37 (3.28–14.44)	Transgastric: 6 (75.0) Transduodenal: 2 (25.0)	FCSEMS: 8 (100.0) DP plastic stent used with each metal stent	NR
Rana 2020	Retrospective	India	EUS: 14	40.1 ± 11.7	13 (93.0)	Amebic: 8 (57.0) Infected pseudocyst: 3 (21.0) Infected biloma: 2 (14.0) Pyogenic: 1 (7.0)	Left: 11 (78.6) Caudate 3 (21.4)	6.1 ± 1.1	Transgastric: 10 (71.4) Transesophageal: 4 (28.6)	DP plastic stents 14 (100.0)	NR
Carbajo 2019	Retrospective comparative	Spain	EUS: 9 PCD: 27	EUS: 66±16 PCD: 66.9±15.0	NR	NR	EUS: Right 5 (55.5), Left 4 (45.5) PCD: Left 22 (81.5), Right 5 (18.5)	EUS: 6.55 (5.1–8.0) PCD: 8.0 (6.0–9.75)	NR	LAMS: 2 (22.2) FCSEMS: 5 (55.5) NCD: 1 (11.1) DP stents used with metal stents	NR
Ogura 2026*	Retrospective comparative	Multicenter	EUS: 43 PCD: 235	EUS: 71 (53–90) PCD: 76 (21–93)	EUS: 25 (58.1) PCD: 160 (68.1)	EUS: Pyogenic 33 (76.7), Iatrogenic 4 (9.3), other (4.7), Idiopathic 18 (41.9) PCD: Pyogenic 85 (36.2), Iatrogenic 9 (3.8), Other 5 (2.1), Idiopathic 137 (58.3)	EUS: Left 29 (67.4), Right 14 (32.6) PCD: Left 89 (37.9), Right 146 (62.1)	EUS: 7.79±1.88 PCD: 7.84±1.70	Transduodenal: 43 (100.0)	Metal (FCSEMS or PCSEMS): 25 (58.1) DP plastic stent: 12 (28.0) NCD: 6 (13.9)	EUS: 18.0±3.8 PCD: 15.2±10.2
Noh 2010	Retrospective	Korea	EUS: 3	59.7±7.8	3 (100.0)	Pyogenic 3 (100.0)	Left 1 (33.3) Caudate 2 (66.6)	5.03±4.67	Transgastric 2 (66.6) Transduodenal 1 (33.3)	NCD: 1 (33.3) DP plastic 3 (100.0)	NR
Chandra 2025	Retrospective	India	EUS: 46	41.5±18.9	44 (95.7)	Amebic 43 (93.5) Pyogenic 3 (6.5)	Caudate 10 (21.7) Left 22 (47.8) Right: 14 (30.5)	6.55±0.33	Transgastric 22 (47.8) Transduodenal 17 (36.9) Transesophageal 7 (15.2)	NCD 31 (67.4)** Aspiration 15 (32.6)	NR
Tanikawa 2023	Retrospective	Japan	EUS: 8	79.5±8.0	4 (50.0)	NR	NR	9.76±4.80	Transgastric 6 (75.0) Transduodenal 2 (25.0)	DP plastic: 7 (78.5) Aspiration: 1 (12.5)	27.5 (24–34)*
DeLa Serna 2014	Retrospective	Spain	EUS: 4	64.75±4.0	2 (50.0)	Pyogenic 4 (100.0)	Left 3 (75.0) Right 1 (25.0)	8.03±1.76	Transgastric 2 (50.0) Transduodenal 2 (50.0)	LAMS: 3 (75.0) CSEMS: 1 (25.0)	NR
Shahid 2023	Retrospective comparative	Multicenter	EUS: 30 PCD: 44	EUS: 69.6. PCD: 60.1	EUS: 13 (43.0) PCD: 20 (45.5)	EUS: pyogenic 25 (83.3), other 5 (16.7) PCD: pyogenic 41 (93.2), Other 3 (6.8)	EUS: left 30 (100.0) PCD: Left 29 (65.9), right 15 (34.1)	EUS: 8.45±6.0 PCD: 7.3±5.5	Transgastric: 27 (90.0) Transduodenal: 3 (10.0)	LAMS: 30 (100.0)	EUS: 54.5 PCD: 51.5
Tonozuka 2015	Retrospective	Japan	EUS: 13	66.9 (19–94)	7 (53.8)	Pyogenic: 7 (53.8) Infected biloma: 6 (46.7)	Left 11 (78.6) Right 3 (21.4)	7.0 (2.2–18.1)	Transgastric 11 (84.6) Transduodenal: 1 (7.7) Transjejunal: 1 (7.7)	FCSEMS: 13 (100.0) with NCD used	25.0 (17.0–43.0)

EUS: endoscopic ultrasound; PCD: percutaneous catheter drainage; N: number; yrs: years; NR: not reported; cm: centimeter; LAMS: lumen-apposing metal stent; FCSEMS: fully covered self-expandable metal stent; PCSEMS: partially covered self-expandable metal stent; DP: double pigtail; NCD: naso-cystic drainage.

* For the study by Ogura et al., the reference year of 2026 was used in the tables and figures, as the article was published online ahead of print in November 2025.

**Table 2:** Events of primary and secondary outcomes of studies included in the final analysis.

Study	Technical success, *N* (%)	Clinical success, *N* (%)	Total adverse events, *N* (%)	Abscess relapse, *N* (%)	Time to stent/NCD removal	Number of EUS sessions	Length of hospital stay	Follow up
Shah 2023	8 (100.0)	7 (87.5)	0 (0)	NR	3 weeks	NR	NR	NR
Ogura 2016	EUS: 8 (100.0)	EUS: 8 (100.0)	EUS: 0 (0)	EUS: 0 (0)	NR	1.5 (1–2)	EUS: 21 (11–200)	EUS: 218 (17–396) days
	PCD: 19 (100.0)	PCD: 17 (89.0)	PCD: 3 (15.8)	PCD: 1 (5.3)			PCD: 41 (17–187)	PCD: 268 (17–1081) days
Rana 2020	14 (100.0)	14 (100.0)	0 (0)	0 (0)	6 weeks	*n* = 1 with 2 EUS sessions	NR	6–106 months
Carbajo 2019 ^2^	EUS: 7 (77.8)	EUS: 7 (77.8)	NR for LAD cases	EUS: 0 (0)	NR	NR	NR	NR
				PCD: NR for LAD				
	PCD: NR for LAD	21 (77.8)						
Ogura 2026	EUS: 42 (97.7)	EUS: 42 (97.7)	EUS: 0 (0)	EUS: 2 (4.7)	At the time of clinical success	NR	EUS: 18.0 (2.0–25.5)	EUS: 141.0 (56.5–502.5) days
	PCD: 233 (99.1)	PCD: 215 (71.5)	PCD: 24 (10.2)	PCD: 12 (5.0)			PCD: 26.0 (17.0–36.5)	PCD: 161.0 (54.0–575.5) days
Noh 2010	3 (100.0)	3 (100.0)	0 (0)	0 (0)	Metal stent: 2 wks Plastic month: 3 months NCD: 5 days	1 (all patients)	NR	7.6 months
Chandra 2025	46 (100.0)	46 (100.0)	8 (25.8)	0 (0)	4–6 weeks	*N* = 3 (aspiration group) 2 EUS sessions	7.6 days	560 (140–1100) days
Tanikawa 2023	7 (87.5)	7 (87.5)	NR	NR	NR	NR	NR	NR
DeLa Serna 2014	4 (100.0)	4 (100.0)	0 (0)	0 (0)	8.5 (4–12) wks	NR	NR	21.5 (4–64) wks)
Shahid 2023	EUS: 30 (100.0)	EUS: 30 (100.0)	EUS: 5 (17.0)	EUS: 2 (4.0)	NR	2.0	EUS: 10.8	EUS: 36.7 wks
	PCD: 44 (100.0)	PCD: 44 (100.0)	PCD: 13 (30.0)	PCD: 4 (9.1)			PCD: 16.0	PCD: 37.0 wks
Tonozuka 2015	13 (100.0)	13 (100.0)	0 (0)	0 (0)	22 (7–60) days ( *n* = 7 patients)	*n* = 1 with 2 EUS sessions	NR	83.5 (24–396) days

EUS: endoscopic ultrasound; PCD: percutaneous catheter drainage; N: number; yrs: years; NR: not reported; wks: weeks; NCD: naso-cystic drainage.

### 3.2 Risk-of-bias Assessment

The four comparative studies were assessed using the ROBINS-I tool and classified as having a moderate risk of bias. The remaining seven single-arm studies were assessed using the NIH Quality Assessment Tool for Before-After (Pre-Post) Studies with No Control Group. Of these, one study 21 was rated as good quality, while the remaining six 16 18 20 22 23 25 were rated as fair, primarily owing to small sample sizes, unclear eligibility criteria, absent consecutive enrollment reporting, and lack of pre-to-post statistical testing. Detailed RoB assessments are provided in **Supplementary Tables 2 and 3** .

### 3.3 Meta-analysis of Outcomes

#### 3.3.1 Clinical Success

**Fig. 1:**
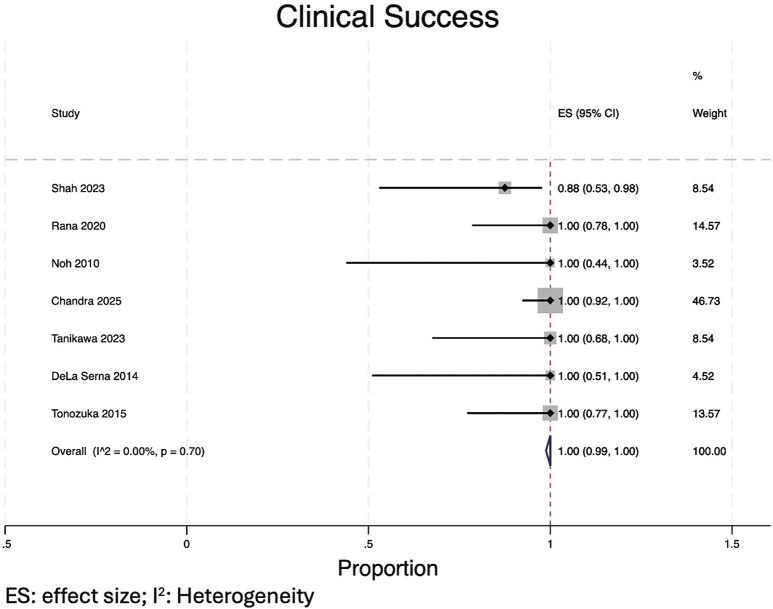
Clinical success of EUS-guided drainage of liver abscess among single-arm studies.

**Fig. 2:**
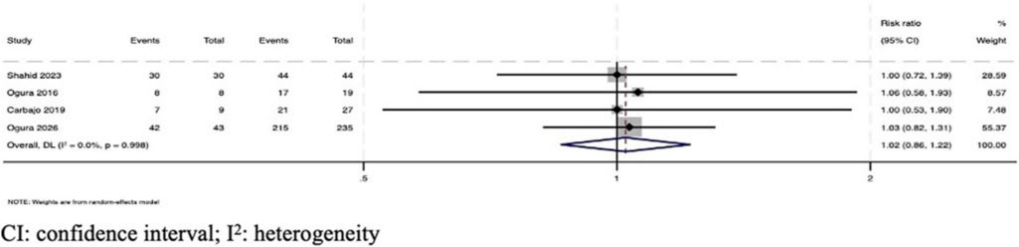
Comparison of clinical success between EUS-guided and percutaneous approach.

Clinical success of EUS-guided abscess drainage was assessed in 11 studies. Among seven single-arm studies, the pooled weighted success rate was 1.00 (95% CI: 0.99–1.00, *I* ^2^ = 0.00%) ( Fig. 1 ). In four studies comparing EUS-guided and percutaneous drainage, no statistically significant difference in clinical success was identified between the two approaches (RR 1.02, 95% CI: 0.86–1.22 ( Fig. 2 ), with no heterogeneity among studies ( *I* ^2^ = 0%).

#### 3.3.2 Secondary Outcomes

##### 3.3.2.1 Technical Success

**Fig. 3:**
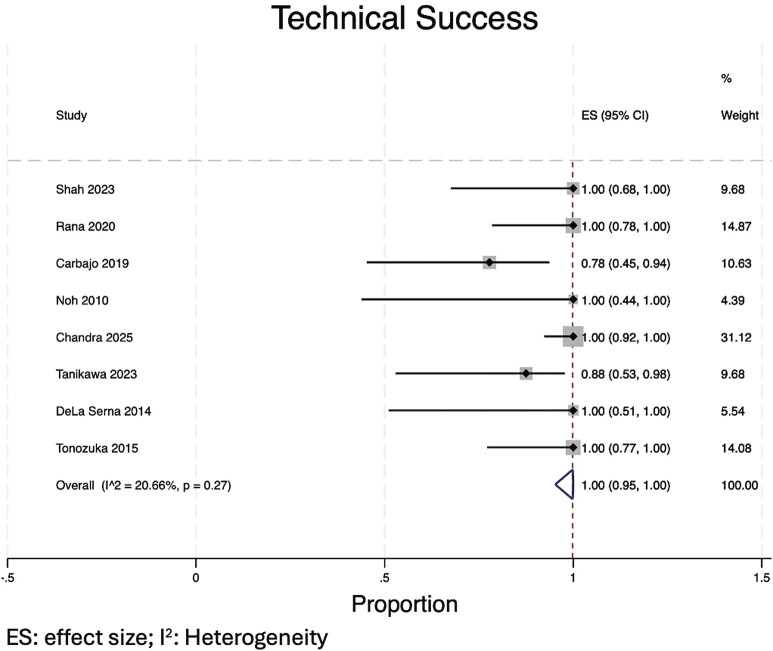
Technical success of EUS-guided drainage of liver abscess among single-arm studies.

**Fig. 4:**
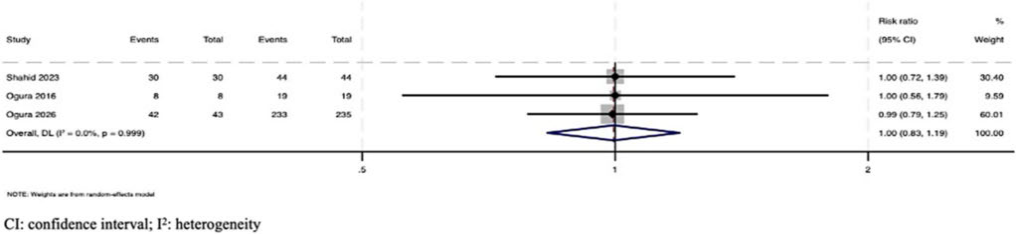
Comparison of technical success between the EUS-guided and percutaneous approach.

Eleven studies evaluated the technical success of the EUS-guided approach. The analysis of eight single-arm studies yielded a pooled weighted success rate of 1.00 (95% CI: 0.95–1.00) ( Fig. 3 ). Low between-study heterogeneity was observed ( *I* ^2^ = 20.66%). In the three comparative studies, the analysis showed no statistically significant difference between the two methods (RR 1.0, 95% CI 0.83–1.19, *I* ^2^ = 0%) ( Fig. 4 ) **.**

##### 3.3.2.2 Total Adverse Events

**Fig. 5:**
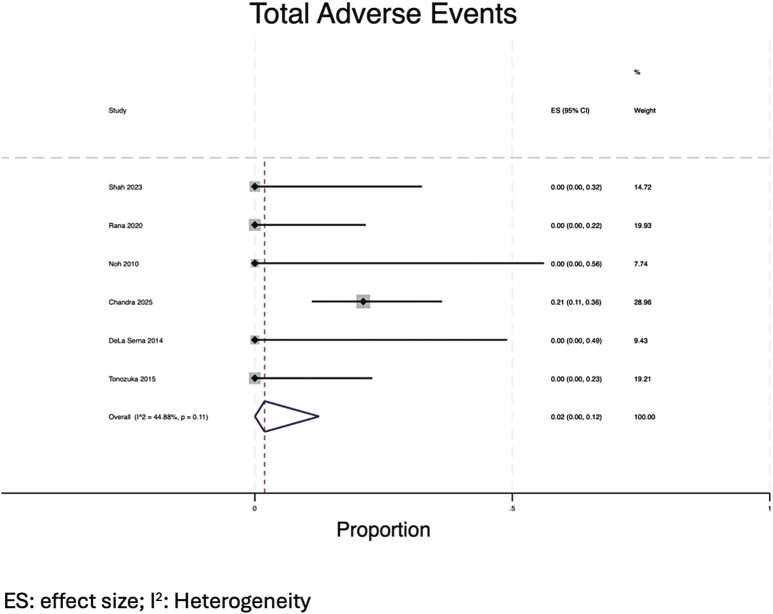
Total adverse event rate of EUS-guided drainage of liver abscess among single-arm studies.

**Fig. 6:**
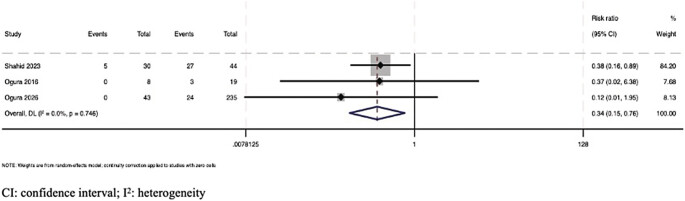
Comparison of total adverse event rate between the EUS-guided and percutaneous approach.

Nine studies reported total adverse events among patients undergoing EUS-guided liver abscess drainage, with 16 events documented. The meta-analysis of six single-arm studies yielded a pooled weighted adverse event rate that was 0.02 (95% CI: 0.00–0.12, *I* ^2^ = 44.88%) ( Fig. 5 ). Three studies directly compared adverse event rates between EUS-guided and percutaneous drainage. The final analysis demonstrated a significantly lower risk of complications with the EUS-guided approach (RR 0.34, 95% CI: 0.15–0.76, *p* = 0.008) ( Fig. 6 ), with no observed heterogeneity among studies ( *I* ^2^ = 0%).

##### 3.3.2.3 Abscess Relapse

**Fig. 7:**
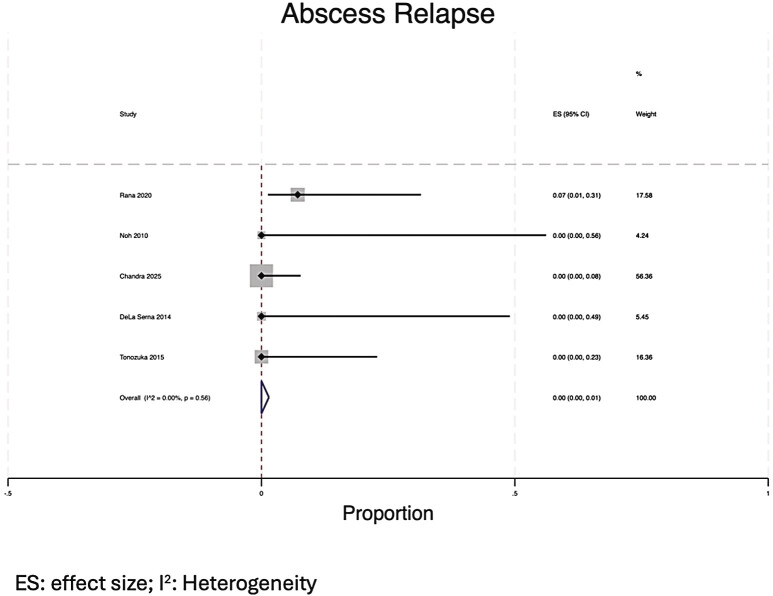
Abscess relapse rate after an EUS-guided drainage of liver abscess among single-arm studies.

**Fig. 8:**
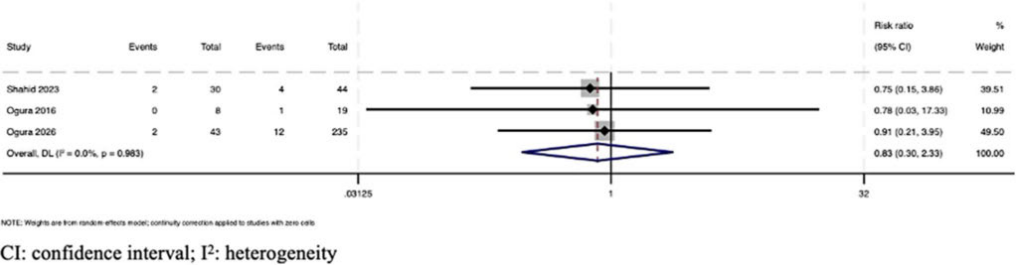
Comparison of abscess relapse rates between the EUS and percutaneous approach.

Abscess relapse was evaluated in eight studies, with relapse occurring in five patients. The pooled weighted relapse rate from the analysis of five single-arm studies was 0.00 (95% CI: 0.00–-0.01, *I* ^2^ = 0%) ( Fig. 7 ). In a meta-analysis of three comparative studies, no significant difference in relapse rates was observed between EUS-guided and percutaneous drainage (RR 0.83, 95% CI: 0.30–2.33), and no between-study heterogeneity was detected ( *I* ^2^ = 0%) ( Fig. 8 ).

### 3.4 Sensitivity Analysis

Sensitivity analyses were performed for all outcomes to assess the robustness of the primary estimates to the method used for handling zero-event cells. For technical success, the risk difference analysis confirmed no statistically significant difference between EUS-guided and percutaneous drainage (log RR 0.00, 95% CI −0.19 to 0.18; *I* ^2^ = 0.0%, *p* = 0.96), consistent with the primary analysis. For clinical success, the risk difference analysis similarly demonstrated no significant difference between the two approaches (log RR 0.02, 95% CI −0.15 to 0.20; *I* ^2^ = 0.0%, *p* = 0.79). For total adverse events, the treatment-arm continuity correction analysis confirmed that EUS-guided drainage was associated with significantly fewer adverse events compared to percutaneous drainage (log RR −1.00, 95% CI −1.86 to −0.15; *I* ^2^ = 0.0%, *p* = 0.02), consistent with the primary analysis. For abscess relapse, the treatment-arm continuity correction analysis demonstrated no statistically significant difference between the two groups (log RR −0.21, 95% CI −1.29 to 0.88; *I* ^2^ = 0.0%, *p* = 0.71). Across all outcomes, sensitivity analyses yielded results consistent with the primary analysis, supporting the robustness of the pooled estimates. Detailed results of the sensitivity analyses are presented in **Supplementary Figs. S2–S5** .

## 4 Discussion

In this systematic review and meta-analysis of 11 observational studies including 511 patients, we comprehensively assessed the efficacy and safety of EUS-LAD not only as a stand-alone approach but also in comparison with PCD. Our results demonstrated that EUS-LAD is technically and clinically successful in approximately all the patients as a single-modality approach with a favorable adverse event and abscess relapse profile. When compared to PCD, although there was no statistically significant difference in technical and clinical success between the two approaches, EUS-LAD was associated with significantly lower risk of complications compared to PCD.

PCD remains the first-line interventional option for the drainage of liver abscess with its overall efficacy ranging from 85% to 97%. 27 One of the main advantages that the EUS approach brings is the elimination of the need for an external drainage, which can be associated with significant complications and impairment of quality of life including leakage, pain, accidental dislodgement, and pleural injury with abscess formation. 6 Additionally, Ogura et al in a retrospective observational study have observed a significantly higher clinical success and shorter hospital stay in patients undergoing EUS-LAD compared to PCD. 7 However, given the paucity of available studies, we were not able to analyze these outcomes in our meta-analysis and would be a field of further research in the future.

The pooled rate of technical and clinical success for EUS-LAD in our meta-analysis was demonstrated to be 100%. However, we should be cautious when analyzing this result. In most of the patients in our study, hepatic abscesses were mostly in the left lobe, which made them more accessible and technically feasible for an EUS approach. Ogura *et al.* evaluated the feasibility of using novel PCSEMS in EUS-LAD to target right-sided hepatic abscesses and achieved a 78.9% technical success rate, which was lower compared to the rest of the included studies. 8 In the three cases with a technical failure of the procedure, the fluid collections were located in segments VII and VIII. 8 Similarly, in another retrospective study comparing the EUS and percutaneous approach, one of EUS technical failures was in a patient with an inaccessible abscess in segment VIII. 19 Thus, hepatic abscesses in the right lobe, and especially in segments VII and VIII, remain technically challenging due to their distance from the gastrointestinal lumen, and in these cases PCD may be considered as a first-line approach. In contrast, liver abscesses located in the caudate lobe pose a significant technical challenge for percutaneous drainage because of the deep anatomical location of the caudate lobe and its proximity to major vascular and biliary structures. 28 In this scenario, the EUS approach offers the advantage of direct visualization and localization of the abscess, facilitating the internal drainage of the abscess with high technical success and an acceptable safety profile. 23

One of the most critical aspects of the EUS approach includes stent selection based on patient’s characteristics and local anatomical factors. Metal stents (including FCSEMS, PCSEMS, and LAMS) have the advantage of larger diameter achieving better drainage of the fluid collections compared to plastic stents and PCD. 19 29 30 However, there are some technical aspects that should be considered before the selection of a metal stent. Firstly, although LAMS are designed to overcome migration issues with bilateral anchoring flanges, they require precise steps to achieve accurate stent deployment. 31 LAMS mis-deployment is one of the most severe complications resulting in full thickness organ defects and perforation requiring high expertise in their closure. 31 Additionally, location of the fluid collection is an important limiting factor, as hepatic abscesses located in the right liver lobe (>1–2 cm from stomach or duodenum) are not suitable for drainage with LAMS. 32 On the other hand, one of the most significant complications with FCSEMS is stent migration, especially in the early postdeployment period, which can occur in 2.5–6.2% of patients undergoing EUS-guided biliary drainage. 33 Overall, in our study, we did not observe events such as stent migration or mis-deployment, a finding that can be explained by the careful selection of patients undergoing EUS-LAD, the high expertise of the endoscopists involved in the cases, and also the use of stents with antimigration function or plastic stents for the stabilization of FCSEMS.

In our study, we were unable to perform a formal cost-effectiveness analysis comparing the two procedures. There is no head-to-head comparison between EUS and percutaneous approach for drainage of liver abscess. In a study by Shahid et al., patients undergoing EUS-guided drainage of liver abscess demonstrated a significantly shorter hospital stay (by 5.2 days; *p* < 0.001), faster symptom resolution (by 10.9 days; *p* < 0.00001), and fewer repeat procedural sessions (mean 2 vs. 7.7; *p* < 0.00001) compared with those in the percutaneous drainage group. 24 Similarly, a retrospective study from Japan reported shorter median hospital stay and fewer adverse events among patients treated with EUS-guided drainage. 17 Although these findings do not represent a direct cost-effectiveness comparison, reductions in hospital length of stay, procedural frequency, and complication rates may translate into lower overall healthcare costs.

The main strength of our meta-analysis includes the comprehensive synthesis of the available literature evaluating the EUS-LAD as a stand-alone approach, but also in comparison with PCD. Although the number of comparative studies was small, we could draw a preliminary conclusion that these two techniques are similar in terms of technical and clinical success, which can render EUS as a safe second-line treatment option, or even as a primary treatment in carefully selected patients.

However, the findings of our meta-analysis should be interpreted with caution and in light of certain limitations. Firstly, all the included studies were retrospective, most of them single-arm, with relatively small sample sies, which may introduce moderate-to-high risk of confounding and selection bias. Given the small number of included studies (fewer than 10 studies), formal assessment of publication bias and small-study effects (e.g., Egger’s test and funnel plot inspection) were not appropriate. Secondly, the results of our meta-analysis may not be generalizable, as all EUS procedures were performed by highly experienced endoscopists in tertiary care referral centers, potentially overestimating the technical success and under-reporting complications of the procedure. Additionally, the high technical and clinical success could be further influenced by the careful and strict selection of patients undergoing EUS-LAD, as most of the patients were found to have left hepatic lobe abscess, which is more easily accessible with the EUS approach. Lastly, although we found a statistically significant difference in total adverse event rates between EUS and percutaneous approach in favor of the former, this finding was primarily driven by the study by Shahid et al., indicating that the pooled estimate may be disproportionately influenced by a single study and should therefore be interpreted with caution.

## 5 Conclusion

Our systematic review and meta-analysis demonstrated that EUS is an effective and safe option for drainage of liver abscess that is comparable to PCD, especially for lesions located in the left and caudate lobes. Although we found significantly lower risk of adverse events with the EUS method, only three studies compared the two methods, which limits the robustness of this result. Until higher quality data is available, PCD remains first-line, and the EUS approach can be considered for select cases. Future studies should not only focus on direct comparison between these two techniques but also evaluate the role of EUS-guided drainage in patients with right hepatic lobe abscesses and further define optimal stent selection through the inclusion of larger patient populations.

## Supplementary Material

Ergänzendes Material Supplementary Material
