# Supplementary material for: Efficacy and Safety of Endoscopic Ultrasound-Guided Drainage in Liver Abscess Management: A Systematic Review and Meta-Analysis
Source: Endosc Int Open. 2026 Jul 29;14:a29191534. doi: 10.1055/a-2919-1534 (PMC13463920; doi:10.1055/a-2919-1534)
Supplement: Supplementary file 1 [file 10-1055-a-2919-1534_29195501.pdf]

Supplementary Material

Authorship: Spyridon Peppas, MD[1], Farhan Kawsar, MD[2], Jerapas Thongpiya, MD[1], Tara Keihanian, MD MPH[1], Jordan Sparkman, MD[1], Salmaan Jawaid, MD[1], Wasif Abidi, MD[1], Kalpesh Patel, MD[1], Mohamed O Othman, MD[1], Fares Ayoub, MD[1]

Author affiliations:

[1]Section of Gastroenterology, Hepatology & Nutrition, Baylor College of Medicine, Houston, TX, USA

[2]Department of Internal Medicine, Baylor College of Medicine, Houston, TX, USA

Title: Efficacy and Safety of Endoscopic Ultrasound-Guided (EUS-Guided) Drainage in Liver Abscess Management: A Systematic Review and Meta-Analysis

Thieme

Supplementary Table 1. Definitions of primary and secondary outcomes of each study

Study	Technical Success	Clinical Success	Tol AEs	Abscess Relapse
Shah 2023	NR	NR	NR	NR
Shahid 2023	Ability to access and drain the abscess	Complete resolution of clinical symptoms with disappearance or at least a > 50% reduction of the abscess size on repeat imaging	NR	NR
Ogura 2016	Successful placement of the PCD tube or EUS-AD stent.	Complete resolution of clinical symptoms, such as abdominal pain and fever, or decreased inflammation on blood examination within 14 days after each procedure.	Defined according to the ASGE lexicon's severity grading system	Typical symptoms with relevant imaging findings
Carbajo 2019	Ability to access and drain the abscess by placement of a drain, with purulent fluid flowing through it.	Complete resolution of clinical symptoms with disappearance of the lesion or a ≥ 50% decrease in size in at least one imaging technique	Any procedure-related event appearing during the procedure or within 2 weeks after its completion graded according to the ASGE lexicon's severity grading system	Appearance of a new abdominal abscess in patients who had achieved clinical success.
Chandra 2025	Ability to access and drain the abscess.	Complete resolution of clinical symptoms with at least 50% reduction in abscess size on repeat imaging	NR	NR
De La Serna 2014	NR	NR	NR	NR
Noh 2010	NR	NR	NR	NR
Ogura 2026	Successful stent or tube deployment.	Decreasing levels of inflammation on blood examinations or decreased size of the maximum liver abscess within 14 days after each procedure to 50% compared with that before EUS-AD or PTAD, and complete resolution of clinical symptoms, such as abdominal pain and fever.	Evaluated according to the severity grading system of the ASGE lexicon	Typical symptoms such as fever, or abdominal pain with observation of liver abscess at the same site based on CT.
Tanikawa 2023	Successful placement of plastic stents or naso-biliary catheters into the abscess, after puncturing the abscess	Successful placement of plastic stents or naso-biliary catheters into the abscess, after puncturing the abscess	NR	NR

Tonozuka 2015	Successful stent placement.	Disappearance of symptoms, resolution of inflammation on blood examination, or decrease in collection size on imaging	According to the ASGE lexicon's severity grading system	NR
Rana 2020	NR	NR	NR	NR

NR: not reported; PCD: percutaneous catheter drainage; EUS-AD: endoscopic ultrasound abscess drainage; ASGE: American Society of Gastrointestinal Endoscopy; PTAD: Percutaneous Transhepatic Abscess Drainage; CT: computed tomography; AEs: adverse events

Supplementary Table 2. Quality assessment of included studies in the meta-analysis according to Risk of Bias in Non-randomized Studies – of Interventions (ROBINS-I) assessment tool

Study	Confounding	Selection	Classification of interventions	Deviation from intended interventions	Missing data	Measurement of outcomes	Selection of reported results	Overall
Shahid 2023	Moderate	Moderate	Low	Low	Low	Low	Moderate	Moderate
Ogura 2016	Moderate	Moderate	Low	Low	Low	Low	Moderate	Moderate
Carbajo 2019	Moderate	Moderate	Low	Low	Low	Low	Moderate	Moderate
Ogura 2025	Moderate	Moderate	Low	Low	Low	Low	Moderate	Moderate

Supplementary Table 3. Risk of Bias Assessment using the NIH Quality Assessment Tool for Before-After (Pre-Post) Studies with No Control Group

Study (Year)	Study Design	Q1	Q2	Q3	Q4	Q5	Q6	Q7	Q8	Q9	Q10	Q11	Q12	Overall Rating
**Chandra 2025**	Retrospective case series	Y	Y	Y	Y	Y	Y	Y	NA	Y	N	N	NA	**Good**
**De La Serna 2014**	Retrospective case series	Y	Y	CD	CD	N	Y	Y	NA	Y	N	N	NA	**Fair**
**Noh, 2010**	Prospective case series	Y	Y	Y	Y	N	Y	Y	NA	Y	N	N	NA	**Fair**
**Rana, 2020**	Retrospective case series	Y	CD	CD	CD	N	Y	Y	NA	Y	N	N	NA	**Fair**
**Shah, 2023**	Retrospective case series	Y	Y	CD	CD	N	Y	Y	NA	Y	N	N	NA	**Fair**
**Tanikawa, 2023**	Retrospective observational	Y	Y	CD	CD	N	Y	Y	NA	Y	N	N	NA	**Fair**
**Tonozuka, 2015**	Retrospective case series	Y	Y	CD	CD	N	Y	Y	NA	Y	N	N	NA	**Fair**

Abbreviations: Y = Yes (criterion met); N = No (criterion not met); CD = Cannot Determine (unclear or absent reporting); NA = Not Applicable. Q8 (blinding) is inherently not applicable to procedural case series. Q10 (pre-to-post statistical testing), Q11 (interrupted time series), and Q12 (group-level analysis) are not applicable by study design.

Thieme

Supplementary Figure 1. PRISMA flowchart of the study selection [26]

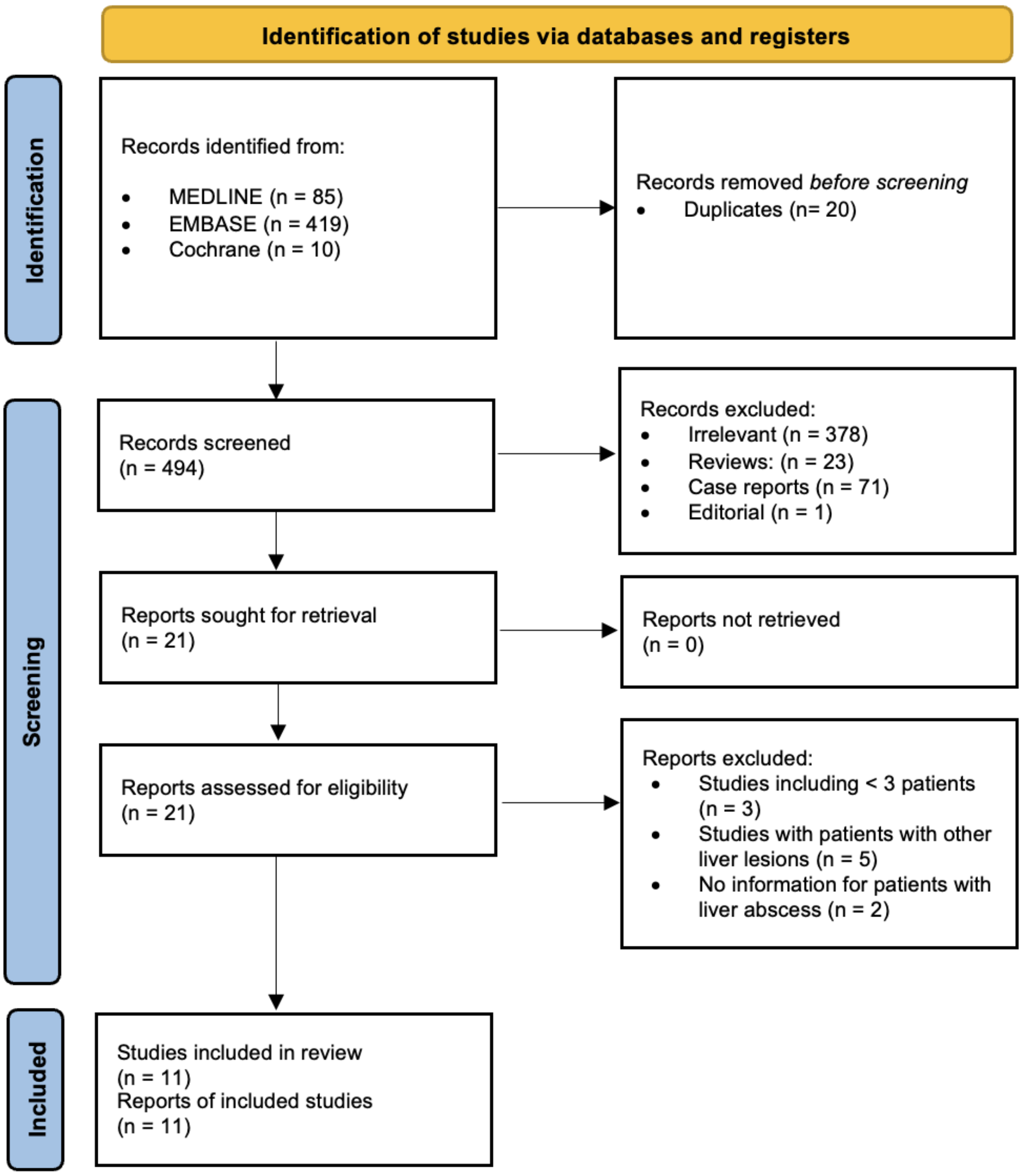

Supplementary Figure 2. Sensitivity analysis using treatment-arm continuity correction for clinical success

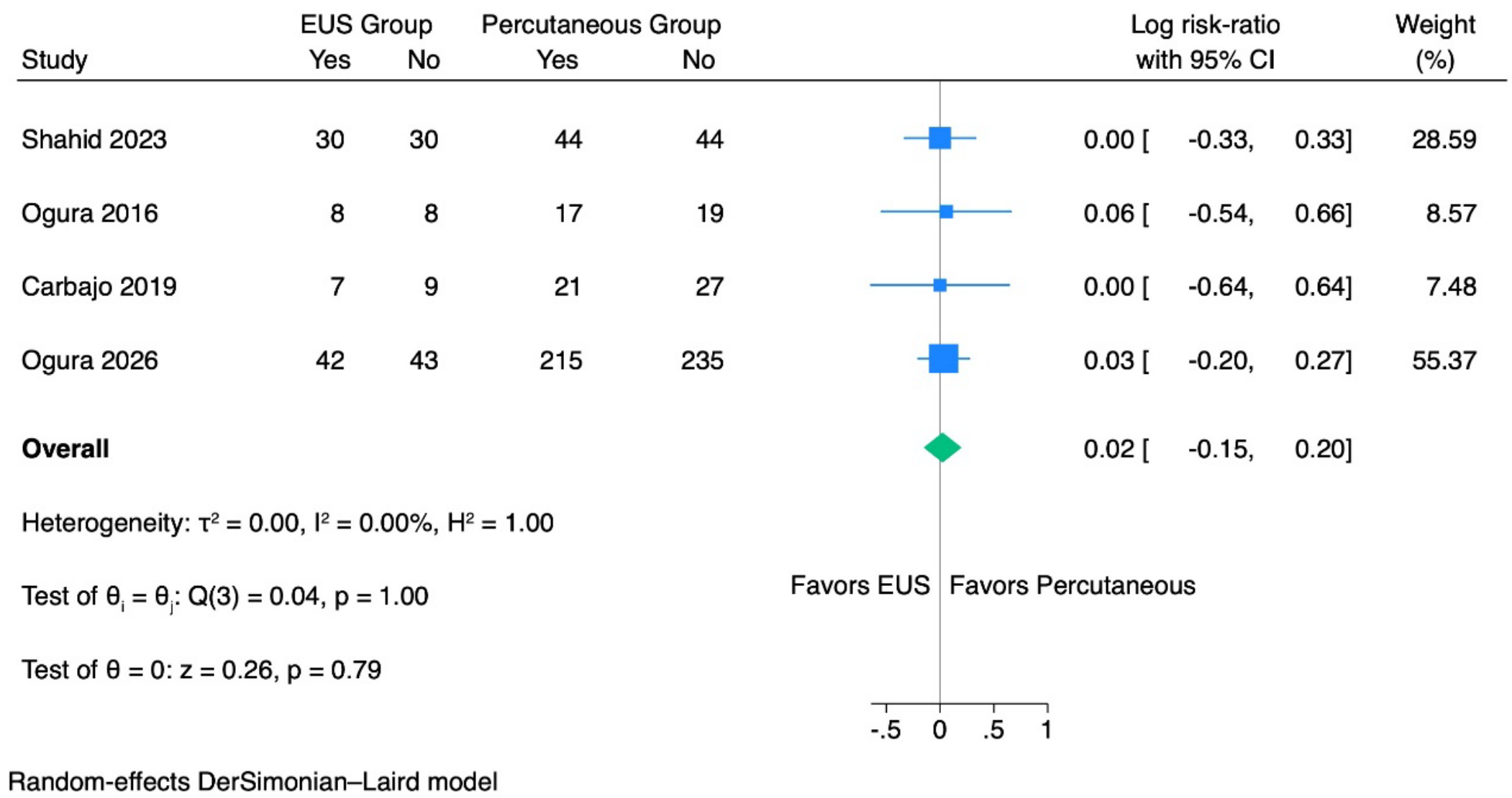

Supplementary Figure 3. Sensitivity analysis using treatment-arm continuity correction for technical success

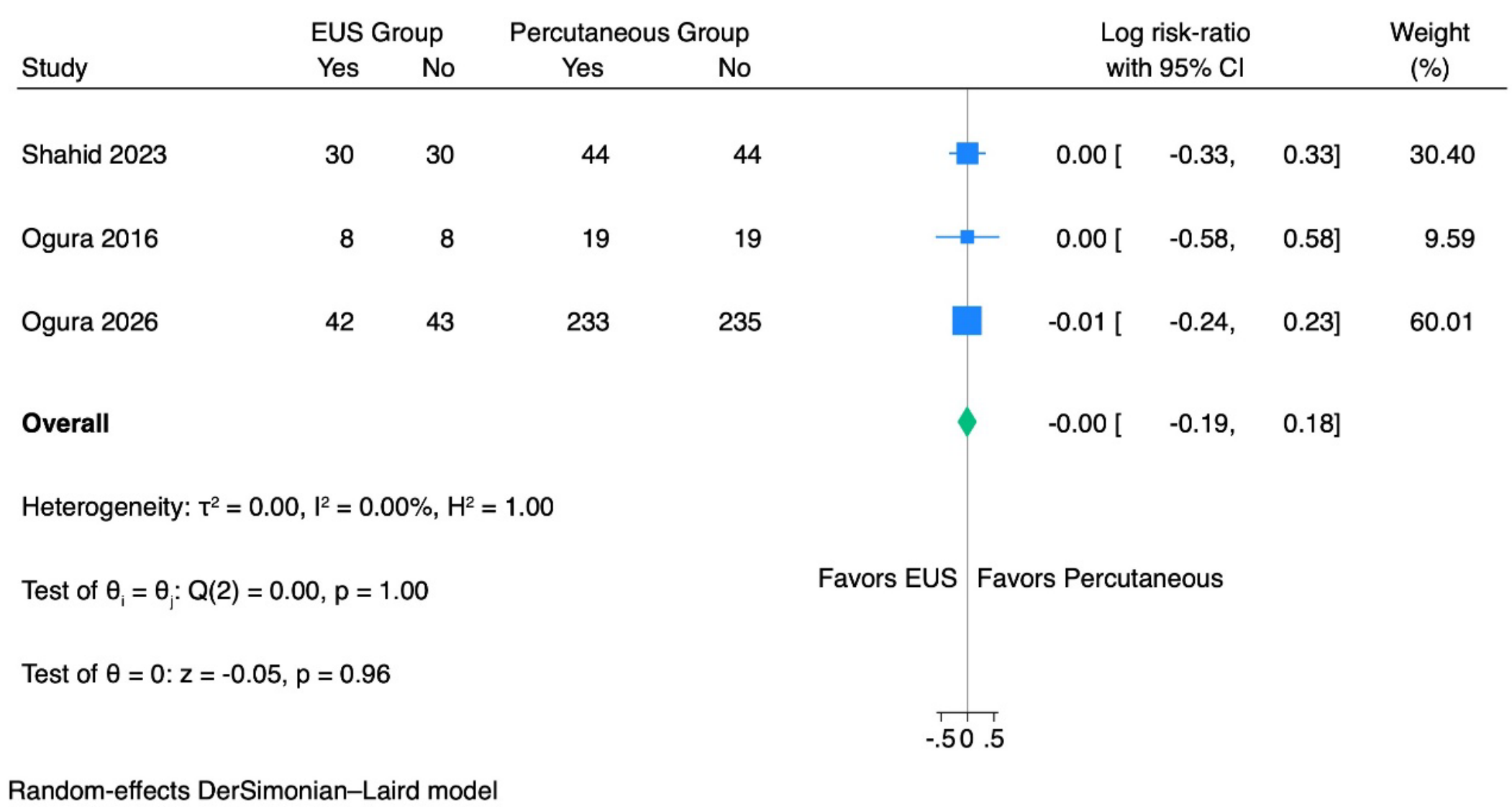

Supplementary Figure 4. Sensitivity analysis using treatment-arm continuity correction for total adverse events

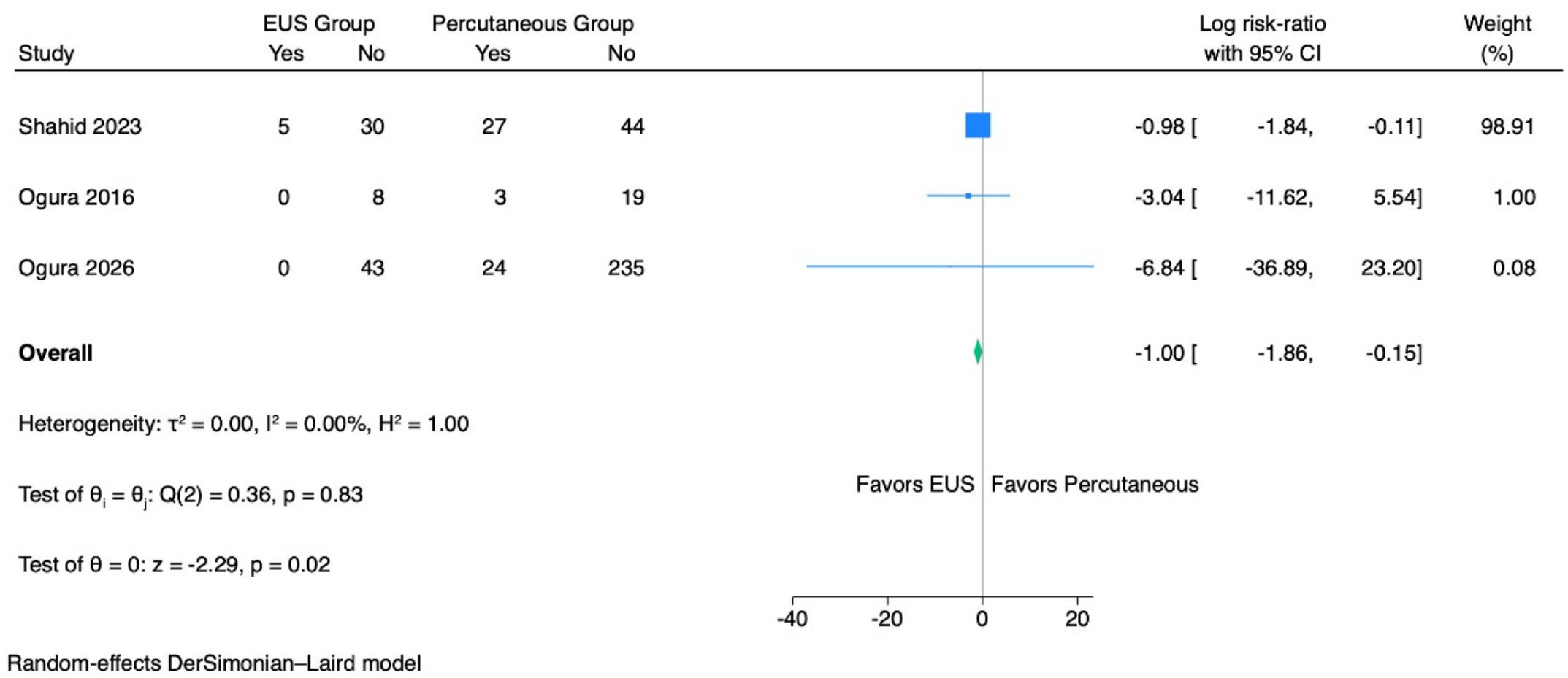

Supplementary Figure 5. Sensitivity analysis using treatment-arm continuity correction for abscess relapse

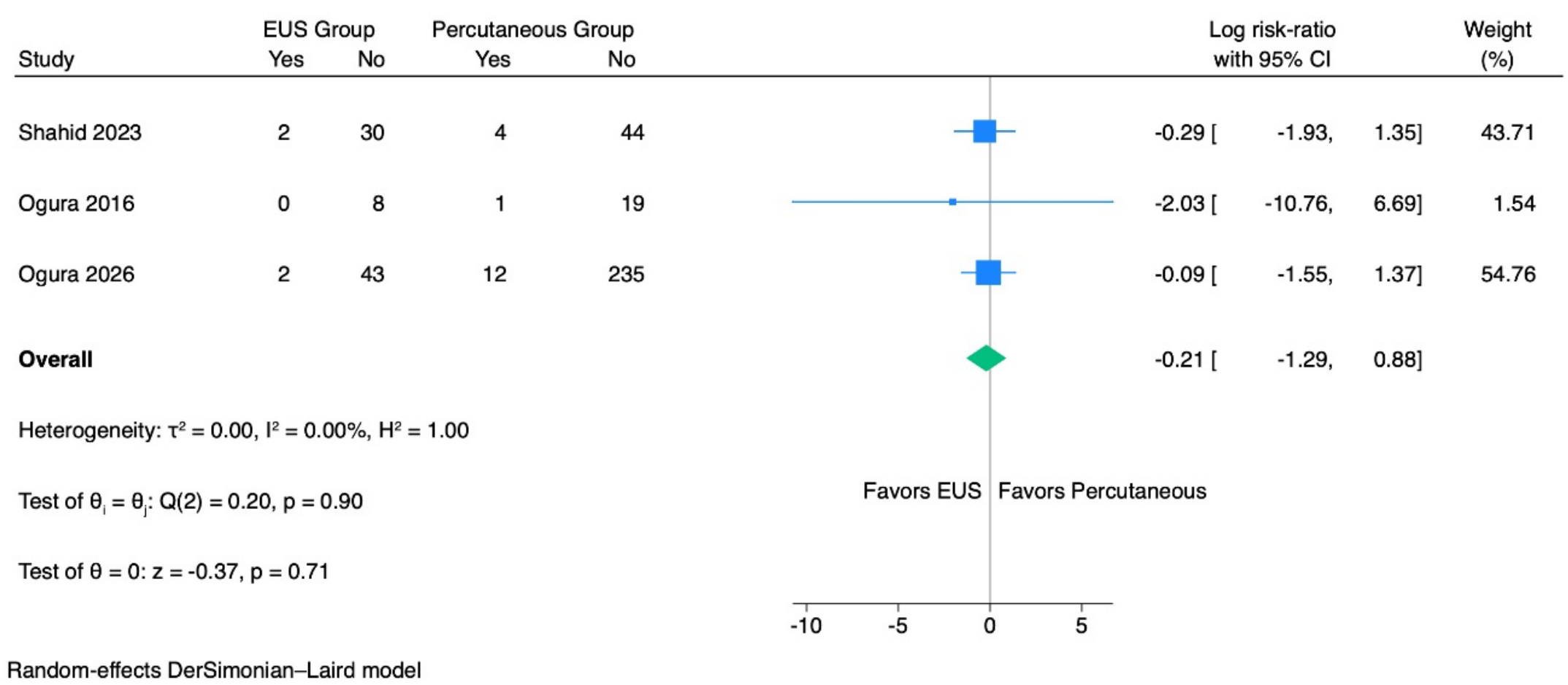

26 Page MJ, McKenzie JE, Bossuyt PM, Boutron I, Hoffmann TC, Mulrow CD, et al. The PRISMA 2020 statement: an updated guideline for reporting systematic reviews. BMJ. 2021 Mar;372:n71.
